# Residual symptoms and functioning in depression, does the type of residual symptom matter? A post-hoc analysis

**DOI:** 10.1186/1471-244X-13-51

**Published:** 2013-02-11

**Authors:** Irene Romera, Víctor Pérez, Antonio Ciudad, Luis Caballero, Miguel Roca, Pepa Polavieja, Inmaculada Gilaberte

**Affiliations:** 1 Department of Clinical Research Lilly, S.A. Avenida de la Industria 30, 28108, Alcobendas, Spain; 2 Autonomous University of Barcelona, Barcelona, Spain; 3 Department of Psychiatry, Hospital Santa Creu i Sant Pau, Autonomous University of Barcelona / CIBERSAM, Barcelona, Spain; 4 Department of Psychiatry, Hospital Puerta de Hierro, Madrid, Spain; 5 Department of Psychiatry, Joan March Hospital, Rediapp, Palma de Mallorca, Spain

**Keywords:** Residual symptoms, Major depression, Functioning

## Abstract

**Background:**

The degrees to which residual symptoms in major depressive disorder (MDD) adversely affect patient functioning is not known. This post-hoc analysis explored the association between different residual symptoms and patient functioning.

**Methods:**

Patients with MDD who responded (≥50% on the 17-item Hamilton Rating Scale for Depression; HAMD-17) after 3 months of treatment (624/930) were included. Residual core mood-symptoms (HAMD-17 core symptom subscale ≥1), residual insomnia-symptoms (HAMD-17 sleep subscale ≥1), residual anxiety-symptoms (HAMD-17-anxiety subscale ≥1), residual somatic-symptoms (HAMD-17 Item 13 ≥1), pain (Visual Analogue Scale ≥30), and functioning were assessed after 3 months treatment. A stepwise logistic regression model with normal functioning (Social and Occupational Functioning Assessment Scale ≥80) as the dependent variable was used.

**Results:**

After 3 months, 59.5% of patients (371/624) achieved normal functioning and 66.0% (412/624) were in remission. Residual symptom prevalence was: core mood symptoms 72%; insomnia 63%; anxiety 78%; and somatic symptoms 41%. Pain reported in 18%. Factors associated with normal functioning were absence of core mood symptoms (odds ratio [OR] 8.7; 95% confidence interval [CI], 4.6–16.7), absence of insomnia symptoms (OR 1.8; 95% CI, 1.2–2.7), episode length (4–24 weeks vs. ≥24 weeks [OR 2.0; 95% CI, 1.1–3.6]) and better baseline functioning (OR 1.0; 95% CI, 1.0–1.1). A significant interaction between residual anxiety symptoms and pain was found (p = 0.0080).

**Conclusions:**

Different residual symptoms are associated to different degrees with patient functioning. To achieve normal functioning, specific residual symptoms domains might be targeted for treatment.

## Background

Residual symptoms of depression cause significant functional impairment [1,2]. This has been reported in patients who respond but are not remitters, partial remitters [1,2], and even in remitters (typically defined as a score of ≤7 on the 17-item Hamilton Depression Rating scale [HAMD-17]) with residual symptoms [2]. Residual symptoms are also associated with persistent functional impairment [1]. However, little is known about the role of specific residual symptom domains, such as core symptoms, symptoms of anxiety, somatic symptoms, and non-painful symptoms.

Most research in patients with residual symptoms has focused on the relationship between residual symptoms and depressive relapse. Several studies have shown an increased risk of relapse [3,4] and rapid relapse [5] in patients with residual symptoms after response without remission. A posthoc analysis from the Sequenced Treatment Alternatives to Relieve Depression [6] study showed that a greater number of residual symptom domains were associated with a higher probability of relapse in full symptomatic remitters. Although a few studies have evaluated the impact of residual symptoms on functional impairment [1,2,7,8], to our knowledge, no published studies have examined the specific role of each residual symptom domain on functional impairment. This is definitely an area worthy of investigation, since the aim of treating depression is not only to achieve clinical remission, but also to return the patient to previous levels of functioning [9]. Knowledge of which residual symptom domains are associated with significant functional impairment and, to what degree would assist physicians in the implementation of specific strategies and treatments to increase the chances of achieving normal or previous levels of functioning.

The aim of this posthoc investigation was to assess the association of specific residual symptoms (core mood, insomnia, anxiety, somatic, and pain) with patient functioning in a large group of patients with an episode of major depressive disorder (MDD) who responded after 3 months of acute antidepressant treatment in routine clinical practice. Our hypothesis was that the strength of the association between residual symptoms and functioning would differ depending on the type of residual symptom.

## Methods

This study is a post-hoc analysis done on a group of MDD patients who responded (improvement of ≥50% on the HAMD-17) after 3 months of acute treatment (n = 624). The analysis of the association between residual symptoms and functioning was done at three months of acute treatment. The source of data was based on a 1-year prospective observational study of a cohort of 930 outpatients with an index MDD episode [10]. As a non-interventional study, the patients were treated according to everyday clinical routine. The protocol was approved by the ethical review board of the Hospital Puerta de Hierro in Madrid, Spain, and all patients provided written informed consent before their inclusion in the study.

### Participants

As described in detail elsewhere [10], adult outpatients with nonpsychotic MDD, single or recurrent episode, according to DSM-IV-TR® [11] were included. Patients had a baseline total score of ≥15 on the HAMD-17) [12] and at least a moderate (≥4) baseline score on the Clinical Global Impression-Severity (CGI-S) scale [13]. Patients suffering from Axis I main psychiatric disorder, dementia, Alzheimer’s disease, organic brain syndrome, or cognitive impairment were excluded from the study.

Patients from this study who responded (improvement of ≥50% on the HAMD-17) after 3 months of acute antidepressant treatment were included in the present analysis.

### Measures and definitions

The HAMD-17 was used to assess the severity of depression and its improvement. Remission was defined as a HAMD-17 score of ≤7 and a response as an improvement of 50% or more from the baseline score. Functioning was measured using the Social and Occupational Functioning Assessment Scale (SOFAS) [14], a 100-point single-item scale used to indicate the individual’s level of social and occupational functioning across a continuum ranging from a state of optimum functioning to a state of important functional impairment. It measures only the level of social and occupational functioning without taking symptoms into account. Thus, a value of 1 represents the hypothetically most impaired individual and 100 the hypothetically healthiest individual. It is completed by a clinician using information from any clinical source. The two highest ranges on the SOFAS, 81–90 and 91–100, describe individuals who not only are without significant psychopathology, but who also exhibit many traits often considered to be components of positive mental health. A SOFAS score ≥80 was used to define normal levels of functioning [15]. The baseline and 3-month follow-up visits were included in this analysis.

Based on Dombrovski [16], we defined the presence of residual core mood symptoms as a score of 1 or more on the HAMD-17 core symptom subscale (depressed mood [Item 1], guilt [Item 2], suicide [Item 3], and anergia/anhedonia [Item 7]). The presence of residual insomnia symptoms was defined as a score of 1 or more on the HAMD-17 sleep subscale insomnia items (early [Item 4], middle [Item 5], and late [Item 6]). The presence of residual anxiety symptoms was defined as a score of 1 or more on the HAMD-17 anxiety subscale (agitation [Item 9], psychic anxiety [Item 10], somatic anxiety [Item 11], and hypochondriasis [Item 15]), and of residual somatic symptoms as a score of 1 or more for item 13 of the HAMD-17.

The Visual Analog Scale (VAS) for Pain was used to assess pain [17], defining the presence of pain as a VAS-overall pain ≥30 mm, which includes patients with at least moderate pain, and had been previously used for the identification of clinically significant pain [18]. The visual analog scale for pain is an instrument widely used in research studies to measure the level of pain. Its simplicity, reliability, and validity, make the VAS the optimal tool for describing pain [19,20]. Pain was measured by the VAS- overall pain, where the patient scores on a 100 point scale the level of overall pain in the last week.

### Analysis

Demographic and clinical data at baseline were described by means of percentages (qualitative variables) or mean ± standard deviation (quantitative variables).

A stepwise logistic regression model was developed to evaluate the association between the residual symptom domains and patient functioning, with a normal level of functioning after 3 months of antidepressant treatment (SOFAS total score ≥80) as the dependent variable, and the following factors as the independent variables in the initial model: Age as a continuous variable, gender, marital status, working status, education status, baseline functioning (SOFAS score), baseline depression severity (HAMD-17), presence of previous episodes of depression, medical co-morbidities, length of current episode, residual symptoms at 3 months (core mood symptoms, insomnia symptoms, anxiety symptoms, somatic symptoms), and pain at 3 months. All the independent variables were included in a full model and then removed stepwise by backward selection (threshold for the p-value = 0.05). Interactions between variables were tested at 3 months (likelihood ratio). Only patients with complete information on the variables previously described were included in the model (n = 600). The reduced model was reported in terms of odds ratios (OR) and their 95% confidence intervals (CI); the fit of the final model was assessed using the Hosmer-Lemeshow goodness-of-fit test [21].

Receiver operating characteristics curves were plotted to determine and compare the sensitivity and specificity of the residual symptom domains and of the pain, as indicators of normal levels of functioning according to the SOFAS (SOFAS score ≥80) after 3 months of treatment. Areas under the curve (AUC) using the trapezoidal rule and their associated asymptotic 95% CIs were calculated. The AUC varies from 0.5 (no apparent accuracy) to 1.0 (perfect accuracy). SAS 9.2 for Windows (SAS Institute Inc., USA) was used for the statistical analysis.

## Results

### Patient disposition, demographics, and clinical characteristics

Of the evaluable sample (N = 930), 624 patients responded to antidepressant treatment and were therefore included in the present analysis. Table 1 shows the socio-demographic and clinical characteristics of the study sample of responders.

**Table 1:** Baseline demographic and clinical characteristics

**Characteristics**	**N = 624**
Age, years, mean (SD)	47.6 (13.8)
Gender, n (%)	
Women	408 (65.4)
Ethnicity, n (%)	
Caucasian	611 (97.9)
Marital status, n (%)	
Married	379 (60.7)
Single	132 (21.1)
Other^1^	113 (18.1)
Education, n (%)	
Illiterate	12 (1.9)
Basic school education	252 (40.4)
High school/higher education	316 (50.6)
Employment status, n (%)	
Active	264 (42.3)
Not working (unemployed or sick leave)	148 (23.7)
Other^2^	212 (34.0)
Patients with medical comorbidity, n (%)	339 (54.3)
Family psychiatric history, n (%)	221 (35.4)
Number of previous episodes, mean (SD)	2.6 (2.5)
Patients with 1st episode, n (%)	280 (44.9)
Age at first episode of depression, mean (SD)	40.5 (14.0)
Current depressive episode:	
Length, mean weeks (SD)	13.9 (23.5)
Chronicity ≥2 years, n (%)	38 (6.1)
HAMD-17 total score, mean (SD)	24.6 (5.7)
Residual symptoms, mean (SD)	
Core mood^3^	8.7 (2.3)
Insomnia^4^	3.9 (1.4)
Anxiety^5^	6.2 (2.2)
Somatic^6^	1.1 (0.6)
VAS-overall pain, total score (mean)	41.4 (28.2)
Monotherapy, n (%)	550 (88.1)
SSRI	145 (26.4)
SNRI	387 (70.3)
Tricyclic antidepressants	6 (1.1)
Others	12 (2.2)
Combination of antidepressants, n (%)	74 (11.9)

MDD = Major depressive disorder; SSRI = Selective serotonin reuptake inhibitor; SNRI = Serotonin-norepinephrine reuptake inhibitor; SD = Standard deviation; VAS = Visual Analog Scale; ^1^Widowed, separated, divorced and other; ^2^Student, housekeeping, retired, disabled worker; ^3^depressed mood [Item 1], guilt [Item 2], suicide [Item 3], and anergia/anhedonia [Item 7] of the HAMD-17; ^4^early [Item 4], middle [Item 5], and late insomnia [Item 6] of the HAMD-17; ^5^agitation [Item 9], psychic anxiety [Item 10], somatic anxiety [Item 11], and hypochondriasis [Item 15] of the HAMD-17; ^6^item 13 of the HAMD-17.

### Residual symptom prevalence

**Figure 1:**
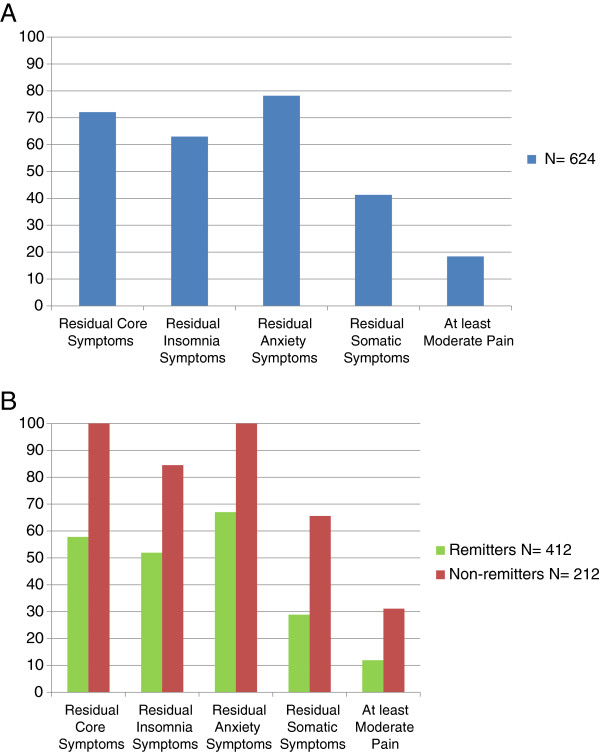
**A Prevalence of residual symptoms domains and pain. **All patients, N = 624. **B **Prevalence of residual symptoms domains and pain, based on remission (HAMD-17 ≤7) N = 412, or non-remission (HAMD-17 >7) N = 212.

After 3 months of acute antidepressant treatment, the most frequent residual symptom was anxiety in 78.2% of patients (95% CI, 74.8–81.4), followed by core mood symptoms in 72.1% (95% CI, 68.4–75.6), residual insomnia in 63.0% (95% CI, 59.1–66.8) and somatic symptoms in 41.3% (95% CI, 37.4–45.3). Pain was reported in 18.4% (95% CI, 15.5–21.7) of patients (Figure 1A). The severity of the residual symptoms and pain was mild (Table 2).

**Table 2:** Residual symptoms, pain and HAMD-17 mean scores after 3 months of acute treatment, N = 624

**Residual symptom**	**Mean (SD)**
Core mood	1.8 (1.6)
Anxiety	1.8 (1.4)
Insomnia	1.0 (0.9)
Somatic	0.5 (0.6)
**Pain**	16.0 (18.4)
**HAMD-17 total score**	6.0 (3.8)

SD = Standard deviation.

After 3 months treatment, 66.0% (412/624) of the patients were in remission (HAMD-17 ≤7). Figure 1B shows the prevalence of residual symptoms by remission status at 3 months. About 90% of remitters (88.3%; 95% CI, 84.8–91.3) had residual symptoms from at least one of the domains studied.

### Residual symptoms and patient functioning

More than half of the patients (59.4%, 371/624) had a normal level of functioning. Factors associated with normal functioning were absence of core mood symptoms (OR 8.7; 95% CI, 4.6–16.7), absence of insomnia symptoms (OR 1.8; 95% CI, 1.2–2.7), shorter episode length (4–24 weeks vs. ≥24 weeks [OR 2.0; 95% CI, 1.1–3.6]) and better baseline functioning (OR 1.0; 95% CI, 1.0–1.1). A significant interaction was found between residual anxiety symptoms and pain (p = 0.0080). The absence of pain increased the chance of normal functioning in either the absence (OR 21.7; 95% CI, 3.5–132.5) or presence of residual anxiety (OR 1.7; 95% CI, 1.0–2.8). However, the absence of residual anxiety was found to increase the chance of normal functioning only if pain was not present (OR 5.2; 95% CI, 2.4.–11.3) (Table 3). Demographic variables, physical co-morbidities, baseline depression severity, previous depression episodes and residual somatic symptoms were not significantly related to functioning (Table 3).

**Table 3:** Association between residual symptoms domains, pain and functioning

	**Odds Ratio**	**95% Wald Confidence Limits**	
**Age**	0.990	0.975	1.005
**Gender (male vs. female)**	1.356	0.886	2.076
**Academic degree (high vs. other)**	1.580	0.949	2.629
**Baseline functioning level (SOFAS)**	1.049	1.031	1.067
**Episode length (4–24 weeks vs. ≥ 24 weeks)**	2.008	1.127	3.579
**Episode lenght (≤4 weeks vs. ≥24 weeks)**	2.138	1.145	3.992
**Absence of residual core mood symptoms**	8.728	4.553	16.730
**Absence of residual insomnia symptoms**	1.796	1.175	2.744
**Absence of residual anxiety symptoms and absence of pain**	5.257	2.445	11.300
**Absence of residual anxiety symptoms and presence of pain**	0.412	0.073	2.341
**Absence of pain and absence of residual anxiety symptoms**	21.669	3.544	132.498
**Absence of pain and presence of residual anxiety symptoms**	1.700	1.025	2.819

Logistic regression model.

SOFAS = Social and Occupational Functioning Assessment Scale;

Dependent variable: normal level of functioning (SOFAS total score ≥80) after 3 months of antidepressant treatment.

The AUC for the residual core mood symptoms was 0.84 (95% CI, 0.80–0.87). Lower AUCs were found for the other residual symptom domains and pain: anxiety 0.75 (95% CI, 0.71–0.78); pain 0.73 (95% CI, 0.69–0.77); insomnia 0.65 (95% CI, 0.60–0.69); and residual somatic symptoms 0.62 (95% CI, 0.58–0.66).

## Discussion

This study evaluated the relationship between patient functioning and specific residual symptom domains (core mood, insomnia, anxiety, somatic symptoms) and pain symptoms in a large group of patients with MDD who responded after receiving acute treatment. Anxiety was the most prevalent residual symptom, followed by core mood symptoms. The strength of the association between the residual symptom domains studied and patient functioning differed depending on the type of symptoms. A more marked association was found for residual core mood symptoms. Residual insomnia was less strongly related to patient functioning, and residual somatic symptoms were not associated.

To our knowledge, this is the first study to investigate the role of specific residual symptoms on patient functioning in MDD. Most publications about residual symptoms in MDD focus on their description and on their relationship to relapse and recurrence of depression [6,22-27]. More recent publications have evaluated their relationship to time to remission [6,28]. Few studies have specifically investigated the relationship between residual symptoms and functional impairment, but instead have focused on the overall impact of these symptoms on functioning without a separate analysis of the type of residual symptom [1,8,29].

As reported previously, residual symptoms are very common after acute treatment, even in remitters [6,22,23]. In the present investigation, we found that almost 90% of remitters had at least one residual symptom domain of mild intensity. This is similar to figures reported by Nierenberg et al. [6] and Ioveno et al. [22]. Similar to other studies, the most common residual symptom domain in our patients was anxiety [6,26]. Other studies have reported residual insomnia [23] and sleep disturbances [6,22] to be the most common residual symptoms domains. These differences may be due to the use of different scales and definitions. Development of a consensus on the definition and measurement of residual symptoms would be desirable to enable results between studies to be compared, thus improving understanding.

Interestingly, we found the strongest association between patient functioning and residual core mood symptoms, and we also found a significant interaction with pain and anxiety. The absence of pain increased the chances of normal functioning, regardless of the presence of residual anxiety. However, the absence of residual anxiety increased the chances of normal functioning only if pain was not present. Of note, we found residual insomnia significantly less strongly related to patient functioning than residual core mood symptoms. In addition, no association was found for residual somatic symptoms. It is remarkable that baseline depression severity and previous depression episodes were not significantly related to functional impairment. This further supports the previous finding that residual symptoms are more important than previous episodes of depression in the prognosis of the patient [5].

The different degree of association of each residual symptom with patient functioning might have prognostic implications and requires further investigation. In line with this, several recent studies tried to identify which specific residual symptoms are predictive of relapse or recurrence [16,26,30,31]. Residual anxiety symptoms were found to be predictive of relapse [26,30,31]. The picture for residual insomnia was less clear, with both positive [16,30] and negative associations reported [6]. Although preliminary, these findings suggest that some residual symptoms present a greater risk for relapse than others.

This study has the following limitations: The primary study from which our data were drawn was not designed to assess residual symptoms, and our results are based on a post-hoc analysis. Our analysis has inherent limitations of post-hoc analysis; measures were those used in the source study. Antidepressant history, before baseline, was not collected, therefore percentage of naïve patients and already treated patients are unknown. This analysis has included a selected population of patients with MDD; patients who had a response to acute antidepressant treatment. This may limit the generalizability of the results to other types of patients not included in this analysis. Our research focused on selected residual symptoms domains and did not include domains such as fatigue or other symptoms not included in the HAMD-17. We also cannot rule out the possibility that a small proportion of the symptoms reported might have been treatment-emergent and not residual.

## Conclusions

In summary, our results contribute to a better understanding of the role of specific residual symptoms domains on functional impairment in depression. We found that different residual symptoms have different degrees of association with patient functioning. This indicates that specific residual symptoms domains may be targets for intervention if normal functioning is the treatment objective.

## Competing interests

Dr. Irene Romera, Dr. Antonio Ciudad, Pepa Polavieja and Dr. Inmaculada Gilaberte are employees of Eli Lilly. Dr. Irene Romera is also an affiliate with the Universidad Autónoma de Barcelona, Departamento de Psiquiatría. Dr. Victor Perez has received grant support from Eli Lilly, Lundbeck, Boehringer, Pfizer, Astra Zeneca, and GSK; has received honoraria from Servier, Eli Lilly, BMS-Otsuka, GSK, Astra Zeneca, and Boehringer; has served as a consultant for and/or on advisory boards for Eli Lilly, BMS, and AstraZeneca. Dr. Luis Caballero has served on advisory boards for Eli Lilly. Dr. Miguel Roca has received grant support from Almirall, Lundbeck and Janssen and served on advisory boards for Eli Lilly and Wyeth.

## Authors’ contributions

IR, PP and IG, have been involved in the analysis. All authors have been involved in the interpretation of the data, decision to submit the manuscript for publication and have read and approved the final manuscript. IR has been involved in writing the manuscript.

## Pre-publication history

The pre-publication history for this paper can be accessed here:

http://www.biomedcentral.com/1471-244X/13/51/prepub

## References

[B1] Romera I Perez V Menchón JM Delgado-Cohen H Polavieja P Gilaberte I Social and occupational functioning impairment in patients in partial versus complete remission of a major depressive disorder episode. A six-month prospective epidemiological study Eur Psychiatry 2010 25 58 65 19553092 10.1016/j.eurpsy.2009.02.007

[B2] Zimmerman M Posternak MA Chelminski I Heterogeneity among depressed outpatients considered to be in remission Compr Psychiatry 2007 48 113 117 10.1016/j.comppsych.2006.10.005 17292700

[B3] Paykel ES Ramana R Cooper Z Hayhurst H Kerr J Barocka A Residual symptoms after partial remission: an important outcome in depression Psychol Med 1995 25 1171 1180 10.1017/S0033291700033146 8637947

[B4] Pintor L Torres X Navarro V Matrai S Gastó C Is the type of remission after a major depressive episode an important risk factor to relapses in a 4-year follow up? J Affect Disord 2004 82 291 296 10.1016/j.jad.2003.11.008 15488260

[B5] Judd LL Akiskal HS Maser JD Zeller PJ Endicott J Coryell W Paulus MP Kunovac JL Leon AC Mueller TI Rice JA Keller MB Major depressive disorder: a prospective study of residual subthreshold depressive symptoms as predictor of rapid relapse J Affect Disord 1998 50 97 108 10.1016/S0165-0327(98)00138-4 9858069

[B6] Nierenberg AA Husain MM Trivedi MH Fava M Warden D Wisniewski SR Miyahara S Rush AJ Residual symptoms after remission of major depressive disorder with citalopram and risk of relapse: a STAR*D report Psychol Med 2010 40 41 50 10.1017/S0033291709006011 19460188 PMC5886713

[B7] Romera I Pérez V Menchón JM Polavieja P Gilaberte I Optimal cutoff point of the Hamilton Rating Scale for Depression according to normal levels of social and occupational functioning Psychiatry Res 2011 30 133 137 20659770 10.1016/j.psychres.2010.06.023

[B8] Miller IW Keitner GI Schatzberg AF Klein DN Thase ME Rush AJ Markowitz JC Schlager DS Kornstein SG Davis SM Harrison WM Keller MB The treatment of chronic depression, part 3: psychosocial functioning before and after treatment with sertraline or imipramine J Clin Psychiatry 1998 59 608 619 10.4088/JCP.v59n1108 9862607

[B9] Keller MB Past, present, and future directions for defining optimal treatment outcome in depression: remission and beyond JAMA 2003 289 3152 3160 10.1001/jama.289.23.3152 12813121

[B10] Ciudad A Alvarez E Roca M Baca E Caballero L Garcia De Polavieja P Casillas M Valladares A Gilaberte I Early Response and Early Remission as Predictors of Good Outcome of a Depressive Episode J Clin Psychiatry 2012 73 185 191 10.4088/JCP.10m06314 22053897

[B11] American Psychiatric Association DSM-TR Diagnostic and Statistic Manual of Mental Health Disorders Text Revision, ed. 4 2000 Washington: American Psychiatric Association

[B12] Hamilton M A rating scale for depression J Neurol Neurosurg Psychiatry 1960 23 56 62 10.1136/jnnp.23.1.56 14399272 PMC495331

[B13] Guy W 1976 ECDEU assessment manual for psychopharmacology 1976 Rockville, Md. U. S: Dept. of Health, Education, and Welfare, Public Health Service, Alcohol, Drug Abuse, and Mental Health Administration, National Institute of Mental Health, Psychopharmacology Research Branch, Division of Extramural Research Programs 313 331

[B14] Goldman HH Skodol AE Lave TR Revising axis V for DSM-IV: a review of measures of social functioning Am J Psychiatry 1992 149 1148 1156 1386964 10.1176/ajp.149.9.1148

[B15] Spitzer RL Gibbon M Endicott J Mental Health Status, Functioning and Disability Measures. In Handbook of psychiatric measures Global Assessment Scale (GAS), Global Assessment of Functioning (GAF) Scale, Social and Occupational Functioning Assessment Scale (SOFAS) 2000 Washington DC: American Psychiatric Association: 1st edition. Edited by First MB 96 100

[B16] Dombrovski AY Cyranowski JM Mulsant BH Houck PR Buysse DJ Andreescu C Thase ME Mallinger AG Frank E Which symptoms predict recurrence of depression in women treated with maintenance interpersonal psychotherapy? Depress Anxiety 2008 25 1060 1066 10.1002/da.20467 18781665 PMC2705944

[B17] DeLoach LJ Higgins MS Caplan AB Stiff JL The visual analog scale in the immediate postoperative period: intrasubject variability and correlation with a numeric scale Anesth Analg 1998 86 102 106 9428860 10.1097/00000539-199801000-00020

[B18] Romera I Fernández-Pérez S Montejo AL Caballero F Caballero L Arbesú JÁ Delgado-Cohen H Desaiah D Polavieja P Gilaberte I Generalized anxiety disorder, with or without co-morbid major depressive disorder, in primary care: prevalence of painful somatic symptoms, functioning and health status J Affect Disord 2010 127 160 168 10.1016/j.jad.2010.05.009 20541811

[B19] Katz J Melzack R Measurement of pain Surg Clin North Am 1999 79 231 252 10.1016/S0039-6109(05)70381-9 10352653

[B20] Bodian CA Freedman G Hossain S Eisenkraft JB Beilin Y The visual analog scale for pain: clinical significance in postoperative patients Anesthesiology 2001 95 1356 1361 10.1097/00000542-200112000-00013 11748392

[B21] Hosmer DW Lemeshow S Goodness-of-fit test for the multiple logistic regression model Communication in Statistics – Theory and Methods 1980 9 1043 1069 10.1080/03610928008827941

[B22] Iovieno N van Nieuwenhuizen A Clain A Baer L Nierenberg AA Residual symptoms after remission of major depressive disorder with fluoxetine and risk of relapse Depress Anxiety 2011 28 137 144 10.1002/da.20768 21284066

[B23] McClintock SM Husain MM Wisniewski SR Nierenberg AA Stewart JW Trivedi MH Cook I Morris D Warden D Rush AJ Residual symptoms in depressed outpatients who respond by 50% but do not remit to antidepressant medication J Clin Psychopharmacol 2011 31 180 186 10.1097/JCP.0b013e31820ebd2c 21346613 PMC3677201

[B24] Conradi HJ Ormel J de Jonge P Presence of individual (residual) symptoms during depressive episodes and periods of remission: a 3-year prospective study Psychol Med 2011 8 1 10 10.1017/S0033291710001911 20932356

[B25] Benvenuti A Rucci P Calugi S Cassano GB Miniati M Frank E Relationship of residual mood and panic-agoraphobic spectrum phenomenology to quality of life and functional impairment in patients with major depression Int Clin Psychopharmacol 2010 25 68 74 10.1097/YIC.0b013e328333ee8e 20061961 PMC3387571

[B26] Taylor DJ Walters HM Vittengl JR Krebaum S Jarrett RB Which depressive symptoms remain after response to cognitive therapy of depression and predict relapse and recurrence? J Affect Disord 2010 123 181 187 10.1016/j.jad.2009.08.007 19733912 PMC2860061

[B27] Bertschy G Haffen E Gervasoni N Gex-Fabry M Osiek C Marra D Aubry JM Bondolfi G Self-rated residual symptoms do not predict 1-year recurrence of depression Eur Psychiatry 2010 25 52 57 19695844 10.1016/j.eurpsy.2009.05.009

[B28] Roca M García-Toro M García-Campayo J Vives M Armengol S García-García M Asensio D Gili M Clinical differences between early and late remission in depressive patients J Affect Disord 2011 134 235 241 Epub 2011 10.1016/j.jad.2011.05.051 21676465

[B29] Ozyüksel B Uluğ B [The association between disability and residual symptoms in depressive patients: a 3-month follow-up.] Turk Psikiyatri Derg 2007 18 323 332 18066723

[B30] Dombrovski AY Mulsant BH Houck PR Mazumdar S Lenze EJ Andreescu C Cyranowski JM Reynolds CF Residual symptoms and recurrence during maintenance treatment of late-life depression J Affect Disord 2007 103 77 82 10.1016/j.jad.2007.01.020 17321595 PMC2680091

[B31] Yang H Chuzi S Sinicropi-Yao L Johnson D Chen Y Clain A Baer L McGrath PJ Stewart JW Fava M Papakostas GI Type of residual symptom and risk of relapse during the continuation/maintenance phase treatment of major depressive disorder with the selective serotonin reuptake inhibitor fluoxetine Eur Arch Psychiatry Clin Neurosci 2010 260 145 150 10.1007/s00406-009-0031-3 19572158

